# Composition and Influence of Fibrogenic Niche in Myocardial Fibrosis

**DOI:** 10.31083/RCM44112

**Published:** 2026-01-08

**Authors:** Xiaohe Wang, Liwei Fan, Qiang Yuan, Yuhang Luo, Zhengyi Cheng, Yi Chen, Chen Gong

**Affiliations:** ^1^Department of Pediatrics, The Second Affiliated Hospital of Anhui Medical University, 230601 Hefei, Anhui, China; ^2^Department of Cardiology, The Second Affiliated Hospital of Anhui Medical University, 230601 Hefei, Anhui, China; ^3^Center of Neurology, Beijing Clinical Research Innovation Lab, 100084 Beijing, China; ^4^The Second School of Clinical Medicine, Anhui Medical University, 230601 Hefei, Anhui, China

**Keywords:** fibrogenic niche, myocardial fibrosis, extracellular matrix, fibroblast activation

## Abstract

Myocardial fibrosis represents the initial stage of cardiac failure and is characterized by the accumulation of extracellular matrix proteins. The fibrogenic niche provides a unique microenvironment for myocardial fibrosis and consists primarily of extracellular matrix proteins, various types of cardiac resident cells, inflammatory cells, extracellular vesicles, and soluble factors. Meanwhile, the composition and contents of this microenvironment undergo dynamic changes during the repair of damaged tissues. Several studies have demonstrated that the fibrogenic niche plays a key role in the activation of fibroblasts, the development of inflammation, and the onset of microvascular dysfunction. Studying the fibrogenic niche has emerged as a new method to clarify the mechanisms involved in myocardial fibrosis, and can potentially facilitate the early diagnosis and individualized medical treatment for the disease.

## 1. Introduction

Heart failure is a life-threatening clinical syndrome that affects more than 64 million people worldwide and its prevalence is increasing [1, 2]. Among the many diseases that contribute to heart failure, myocardial fibrosis is the pathophysiologic basis that is closely associated with these diseases and their prognosis. Myocardial fibrosis is a dilatation of the interstitium of the heart due to the accumulation of extracellular matrix (ECM) proteins [3]. It has traditionally been considered irreversible, so identifying, preventing, and treating fibrosis in the clinical setting is an important and daunting task. Current therapeutic options regarding heart failure are relatively well developed [4], but effective treatment options for reversing myocardial fibrosis are still lacking. Therefore, this situation requires us to rethink the mechanisms of myocardial fibrosis and find new targets for intervention.

It is hypothesized that a unique microenvironment exists after tissue injury, and that the composition and contents of this microenvironment change dynamically during the remodeling of the repaired tissue. In particular, extracellular matrix proteins change after injury, which in turn affects the activation status and functional behaviors of adjacent cells, altering their phenotype and trajectory. We elaborated on this idea by coining the term “fibrogenic niche” to define the tissue-specific microenvironment that drives the activation of fibroblasts during organ fibrosis [5]. This concept differs from the broadly defined, diffuse, pathological environment—the pro-fibrotic microenvironment—because it represents a localized, highly specialized functional unit. At its core is the precise anchoring and regulation of “effector cells” that promote fibrosis by directly stimulating the deposition of ECM in tissues.

This concept is meaningful in the description of myocardial fibrosis and could be a novel approach to illuminating the underlying mechanisms governing myocardial fibrosis.

This article provides a review of the fibrogenic niche’s composition, biological functions, and operational mechanisms in myocardial fibrosis. In addition, we examine the potential relevance of the fibrogenic niche hypothesis in the future diagnosis and treatment of fibrotic diseases.

## 2. Major Events in Myocardial Fibrosis

Basic histopathologic analysis classifies cardiac fibrotic lesions into three distinct forms: ‘alternative fibrosis’, ‘interstitial fibrosis’, and ‘perivascular fibrosis’. Staining is used to label collagen fibers to clearly differentiate fibrosis from normal myocardial structures [6].

The reasons for fibroblasts’ differential responses at various sites following the same injury are unknown. There is speculation that a distinct tissue microenvironment might underlie the activation process of fibroblasts at different locations. We term this description of a tissue microenvironment that promotes fibroblast activation in organ fibrosis the fibrogenic niche [5]. The notion has been applied to kidney [7], liver [8] and lung [9] fibrosis. This review describes the fibrogenic niche in cardiac fibrosis caused by chronic diseases.

Myocardial fibrosis is a progressive process: a fibrogenic niche is first established, and this is followed in turn by the deposition and gradual expansion of ECM proteins, which results in extensive fibrosis. During the process, a series of events occur in the fibrogenic niche, such as cardiomyocyte injury, inflammatory cell infiltration, and fibroblast activation. Cardiomyocyte injury leads to the release of inflammatory factors, transformation and the senescence of vascular endothelial cells, which is accompanied by infiltration of inflammatory cells, such as macrophages, and the transformation of fibroblasts to myofibroblasts with high expression of α-smooth muscle actin (α-SMA) [10], which transmit fiber contractility to the ECM [11]. These cells also secrete ECM proteins, extracellular vesicles (EVs), and soluble factors to promote the formation of fibrogenic niches.

## 3. Structural Components of the Fibrogenic Niche

**Fig. 1.**
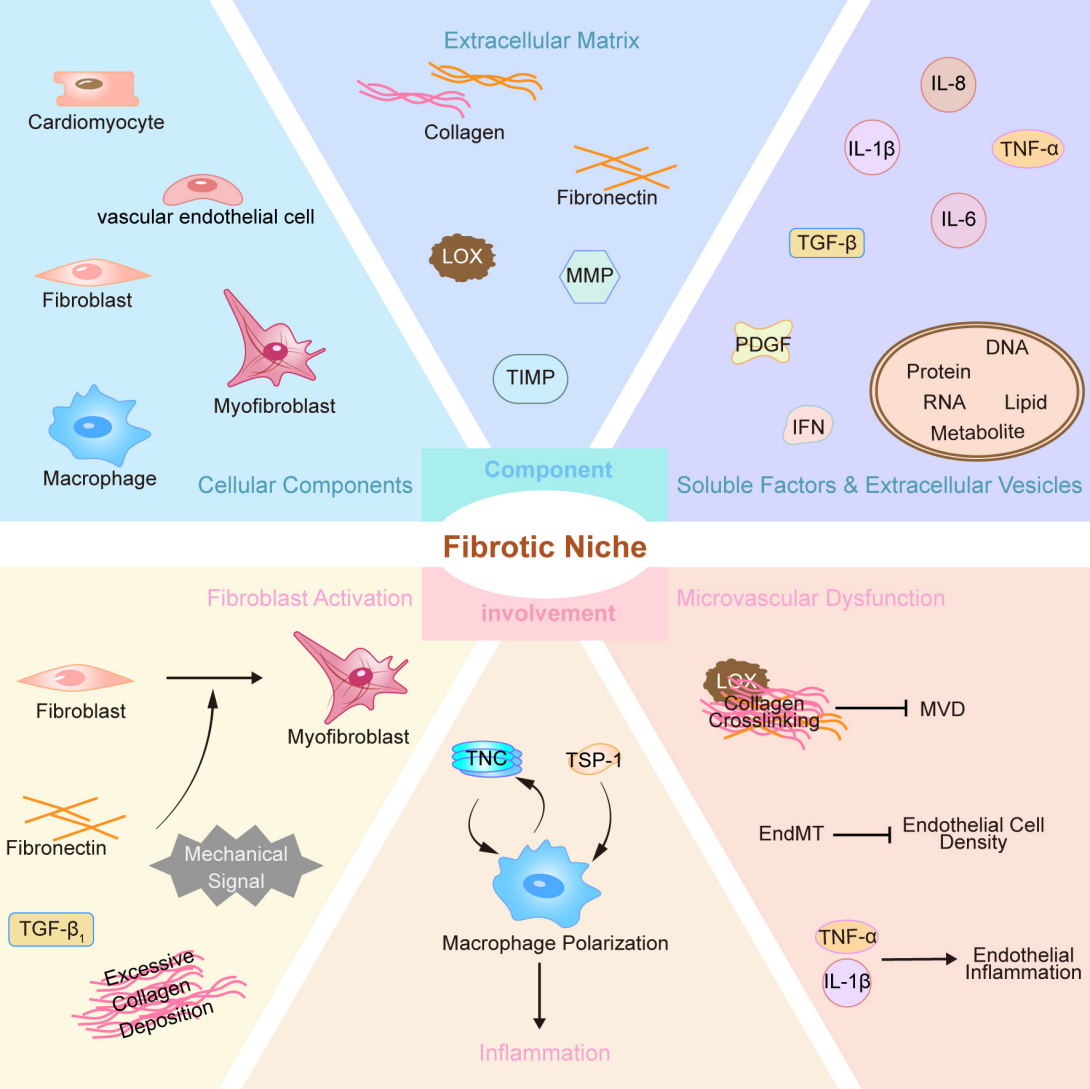
**Major component and involvement of fibrogenic niche in myocardial fibrosis**. PDGF, platelet-derived growth factor; IFN, interferon; LOX, lysyl oxidase; MVD, microvascular density; EndMT, endothelial-mesenchymal transition; TNC, Tenascin-C; TSP-1, Thrombospondin-1; TGF-β, transforming growth factor-β; MMP, matrix metalloproteinases; TIMP, inhibitors of metalloproteinases; DNA, deoxyribonucleic acid; RNA, ribonucleic acid; EVs, extracellular vesicles. Cardiomyocytes: secrete TGF-β1 and TNF-α. EVs: deliver pro-fibrotic factors.

Similar to the well-characterized stem cell niche [12] and the renal fibrogenic niche [7], the fibrogenic niche of the myocardium consists of a variety of structural components containing cardiac-resident cells (e.g., cardiomyocytes, vascular endothelial cells, myocardial fibroblasts, and vascular wall cells), infiltrating inflammatory cells (e.g., macrophages, mast cells, and dendritic cells), and secreted soluble factors, EVs, and extracellular matrix (Fig. 1).

Among the cardiac resident cells, cardiomyocytes and vascular endothelial cells are the main targets of various injuries, and they have various responses to injury. Examples include cardiomyocyte death [13] and release of danger-associated molecular pattern (DAMP) [14], endothelial-mesenchymal transition [15, 16], senescence, and cell cycle regulation occurring in vascular endothelial cells. Despite the existence of different modes of response to injury, the cells tend to secrete large amounts of pro-inflammatory [17] and pro-fibrotic factors that activate fibroblasts [18]. Cellular senescence leads to the development of a senescence-associated secretory phenotype (SASP), along with the secretion of elevated levels of pro-inflammatory and pro-fibrotic factors—including cytokines, chemokines, proteases, and growth factors [19]. In addition, endothelial cells have paracrine signaling, and interactions with cardiomyocytes [20, 21].

Therefore, very high levels of soluble factors and extracellular vesicles may be present within the fibrogenic niche. These extracellular vesicles and soluble factors make the ECM a special environment for promoting the proliferation of fibroblasts.

In this environment, extracellular vesicles are secreted into the ECM by a variety of cells as a medium for intercellular communication [22]. There are three main types [23]: exosomes, microvesicles and apoptotic vesicles, whose surface receptors [24] and internally carried substances (including proteins, ribonucleic acid (RNA), deoxyribonucleic acid (DNA), lipids and metabolites, etc.) [25]. These are capable of transferring information, for example, they can be bound to the ECM through surface integrins and interactions [26], which can serve as a localization agent, and they can also have an impact on immune cell function [27].

Soluble factors in the fibrogenic niche include IL-1β, IL-6, IL-8, IL-12, TNF-α, and interferon, which promote inflammatory responses and tend to stimulate immunocompetent cells [28], as well as C-C motif chemokine ligand 2 (CCL2), transforming growth factor-β (TGF-β), Wingless-type MMTV integration site family (Wnt), fibroblast growth factor (FGF), and platelet-derived growth factor (PDGF) [6, 29, 30]. When the injury persists, endothelial cells become dysfunctional [31] or even apoptotic [32], resulting in vascular thinning or disintegration in severe cases, leading to ischemia or necrosis of tissues [33] and further propagating fibrotic signals. Beyond creating a suitable setting for fibroblast activation and proliferation, the fibrogenic niche further impairs the function of remaining viable cells and hinders the progression of tissue repair.

The ECM also functions as another major structural element of the fibrogenic niche [34]. It is a mechanical scaffold composed of various proteins (e.g., type I and type III collagen, as well as reservoirs of various glycoproteins, stored potential growth factors, and proteases) that provide structural and biochemical support to the surrounding cells [35] and is also important for the transmission of contractile forces [36]. The various types of substances in the ECM are highly dynamic. For example, after myocardial injury, type I and type III collagen-based structural ECM proteins are overdeposited [37]. The composition of the ECM varies temporally and spatially during cardiac development and repair [38]. Therefore, it is reasonable to speculate that there are specialized ECM networks in the fibrotic state of the myocardium that are used to regulate various cellular behaviors.

The myocardial fibrogenic niche, once defined as restricted to a fixed region, and the reasons for how the location was initially selected and restricted to this region are not clear. The cellular components of the fibrogenic niche are dynamically mobile, and by comparison, extracellular vesicles and soluble factors spread following a concentration gradient—traits that render them ill-suited for localizing and sustaining the fibrogenic niche. Conversely, the ECM network displays a static property: once established at a specific site, its components typically form a well-demarcated region after deposition—a scenario that aligns with the concept of the myocardial fibrogenic niche.

Therefore, we propose that the ECM is a key part of the fibrogenic niche and acts to regulate fibrosis by recruiting cells and secreted factors from the surrounding environment through receptor or non-specific binding, which in turn fixes the position of the ecological niche.

## 4. ECM Components of the Fibrogenic Niche

Because the ECM network is important for fibrogenic niche formation [5], it is also essential to determine its molecular composition and to focus on the role and dynamic changes of its components in the process of myocardial fibrosis. A study [39] analyzed the differences between ECM proteins in myocardial *in vitro*-control and pro-fibrotic groups, and identified a total of 352 matrix components by mass spectrometry (MS) in the isolated decellularized ECM (dECM), broadly categorizing the proteins studied in depth into: structural proteins, basal lamina components, growth factors, and proteins involved in remodeling of the ECM.

The expression and deposition of structural proteins are significantly increased during fibrosis. Hyperactivation of fibroblasts in the heart transforms them into myofibroblasts, which increases the production of type I and type III collagen, yet degradation decreases, consequently, resulting in abnormal collagen deposition [40]. During the advanced phases of the disease, the persistent activation of fibroblasts results in collagen I deposition surpassing its rate of degradation, which eventually gives rise to irreversible fibrosis.

Collagen deposition is a key feature of cardiac fibrosis [41], and in the future, its derived peptides may become biomarkers of myocardial fibrosis and prognostic indicators of heart failure [40]. Its structural components include glycoproteins such as Periostin (POSTN), Fibronectin (FN), Elastin (ELN), and Fibulin 5 (FBLN5). Fibronectin (FN) that deposits rapidly in the early course of the disease recruits inflammatory cells and fibroblasts, thereby initiating tissue repair. Later, it is broken down by matrix metalloproteinases (MMPs) in order to free up space for the deposition of mature collagen, serving as a critical component of the “temporary ECM scaffold”. ELN is a protein that makes tissues elastic and is involved in the progression of cardiovascular diseases in the dECM, which is upregulated in models of fibrosis [42]. In fibrotic ECM, fibroblasts produce POSTN, FN, and FBLN5 which act as integrin ligands and are important for the production collagen fibers [43, 44].

The basal lamina component contains type IV collagen and laminin. Type IV collagen forms the core meshwork of the basal lamina and provides support for cardiomyocytes and vascular endothelial cells, and to some extent it also reflects the degree of fibrosis [45, 46]. The oligopeptide Arginine-Glycine-Aspartic acid (RGD) present on the surface of laminin is recognized by integrins, which in turn have an impact on pathological processes such as cardiac fibrosis and hypertrophy [47].

Growth factors, contain proteins of the TGF-β, fibroblast growth factor (FGF), vascular endothelial growth factor (VEGF), bone morphogenetic protein (BMP) and platelet-derived growth factor (PDGF) families. For example, TGF-β-Smad2/3 signaling underlies cardiac fibrosis [48]. When TGF-β is inhibited, myocardial fibrosis is alleviated and cardiac function is restored [49]. Proteins of the FGF, VEGF, BMP and PDGF families are also involved in the regulation of cardiac function and myocardial fibrosis [50, 51, 52, 53, 54, 55, 56, 57]. The FGF, VEGF, and BMP families of proteins primarily have anti-fibrotic effects, whereas the PDGF family of proteins promotes fibrosis.

The process of myocardial injury is accompanied by dynamic changes in the ECM. ECM remodeling is dependent on matrix metalloproteinases (MMP), inhibitors of metalloproteinases (TIMP), lysyl oxidase (LOX) and LOX-like proteins. MMP promotes tissue remodeling and the renewal of a wide range of ECM proteins including collagen, elastin and other glycoproteins. In addition, TIMP is an endogenous MMP inhibitor that reduces the degradation of ECM proteins [58]. LOX also plays a role in remodeling the ECM [59], and has been associated with the development of myocardial fibrosis and cardiomyocyte apoptosis, which can predict the risk of cardiovascular disease [60].

ECM proteins can form complex matrix structures through direct interactions or LOX-mediated cross-linking, each functioning and reinforcing the structure [61]. For example, collagen rich in hydroxylysine-derived crosslinks (such as type I collagen) exhibits significantly enhanced mechanical strength following sustained activation by LOX and its homologues, and is less susceptible to degradation by matrix metalloproteinases [62]. Transglutaminase (TG2) is a key enzyme regulating ECM cross-linking and cellular activation during myocardial fibrosis. Under pathophysiological conditions, TG2 is highly expressed in blood vessels, where it promotes vascular function and enhances vascular stiffness through both cross-linking-dependent and cross-linking-independent mechanisms [63].

## 5. The Involvement of the Fibrogenic Niche in Fibrogenesis Progression

The ECM network is a key part of the myocardial fibrogenic niche. Components in the ECM, in conjunction with extracellular vesicles and cytokines, establish a localized microenvironment that drives fibroblast activation and proliferation, and also exerts effects on inflammatory cell infiltration and vascular dysfunction (Fig. 1).

### 5.1 Fibroblast Activation

Activated fibroblasts are the central cellular effectors of myocardial fibrosis and are the main cells producing ECM proteins [64]. Fibroblasts are stimulated to transform into myofibroblasts that are capable of secretion, matrix production and contractility [41], which are important factors in the progression of fibrosis.

Activated myofibroblasts are the main source of structural ECM proteins in the fibrotic heart, producing large amounts of stromal cell proteins and also regulating matrix remodeling by producing proteases and protease inhibitors [58]. The majority of research has addressed the mechanisms related to soluble factors (e.g., TGF-β1, etc.) in triggering fibroblast activation [65, 66, 67]. There is also a multitude abundant literature suggesting that changes in the remaining ECM composition and viscoelasticity can also lead to fibroblast activation. In fibrotic ECM, the overdeposition of collagen fibers (in particular type I collagen which make up 90% of fibroblasts) can be mediated through paracellular stretch signaling, which transmits mechanical signals to surrounding cells [68, 69], and promotes fibroblast activation [70]. Fibrotic ECM also enhances α-SMA expression through mechanistic signaling pathways such as integrin-cytoskeleton, TGF-β receptor, and mechano-force-sensitive ion-gated channel-calcium ion-YAP (Yes-associated protein)/TAZ (Transcriptional co-activator with PDZ-binding motif) [69], which is an indication of fibroblast activation. Upregulation of FN expression in fibrotic ECM also promotes fibroblast differentiation to myofibroblasts [71], increasing collagen deposition and remodeling of tissues. These various functions suggest that fibroblast activation and ECM fibrosis are bi-directionally regulated.

### 5.2 Inflammation

Inflammation acts as a critical constituent of myocardial fibrosis, with the fibrogenic niche driving the infiltration of inflammatory cells. For example, Tenascin-C (TNC) enhances the pro-inflammatory phenotype of macrophages, and *in vitro* experiments [72] have demonstrated that TNC accelerates ventricular remodeling by modulating macrophage polarization. Pro-inflammatory cytokines also up-regulate the expression of TNC, which may create a positive feedback that amplifies inflammation [73]. Thrombospondin-1 (TSP-1) also activates macrophages through a TLR4-dependent pathway [74]. In addition, macrophage-to-myofibroblast conversion is present in myocardial fibrosis [75], and may contribute to the establishment of the fibrogenic niche.

### 5.3 Microvascular Dysfunction

Microvascular function becomes dysfunctional during myocardial fibrosis. For example, during myocardial fibrosis, excessive deposition of type I collagen and cross-linking between collagen increase the stiffness of myocardial tissue. There is data which shows that the degree of myocardial fibrosis is negatively correlated with the density of microvessels [76].

As previously noted, MMP is involved in the degradation of ECM proteins, and vascular density during myocardial infarction is increased in MMP-9-deficient mice [77]. A large amount of cytokines are released by various types of cells after myocardial fibrosis (e.g., TNF-α, IL-1β), which initiate an inflammatory cascade response in endothelial cells by activating the TLR4 and NF-κB pathways. The inhibition of TNF-α reduces structural remodeling and improves hemodynamics [78], in addition to inflammatory factors that can reduce endothelial cell density by activating endothelial-mesenchymal transition (EndMT) [79].

## 6. Mechanism of Action of the Fibrogenic Niche

The mechanisms by which the fibrogenic niche influences the behavior of neighboring cells to alter disease progression remain unclear. However, it is established that ECM proteins can anchor cells to specific locations through integrin binding. In addition, due to the cross-linking of collagen fibers in fibrotic myocardium, the fibrogenic ECM typically exhibits greater tissue stiffness compared to normal ECM, and mechanical stress promotes fibroblast activation.

### 6.1 Recruitment and Binding of Soluble Factors

The fibrogenic niche appears to not only attract but also sequester soluble factors derived from the adjacent extracellular compartment, establishing a microenvironment characterized by elevated concentrations of profibrotic factors and signaling molecules. TNC may be involved in this process, as it associates with multiple profibrotic factors with strong affinity. A study [80] has shown that TNC is a hexameric protein with a multi-domain structure, enabling it to bind various ECM components and cell types due to its unique architecture. Its FNIII interacts with multiple proteins, including integrins, contactins, annexin II, as well as growth factors such as FGF, PDGF, and TGF-β, indicating that TNC is critical for cell adhesion and signaling pathways [81, 82].

Soluble factor-sequestering and -binding ECM proteins may further exert a key function in delivering various ligands toward the homologous cell membrane receptors. In contrast to free soluble factors, ECM proteins bound to high concentrations of cytokines can more efficiently transmit information to the corresponding cell membrane receptors, thereby triggering receptor activation and the subsequent signaling.

### 6.2 Activation of Signaling Pathways

ECM proteins in the myocardial fibrogenic niche can bind to different receptors to activate distinct signaling pathways. The known receptors include integrin, Wnt and TLR, through which cells within the fibrogenic niche can be activated by ECM components.

#### 6.2.1 Integrins

Integrins represent a major category of classic ECM receptors, composed of α/β heterodimers that are assembled from 18 α-subunits and 8 β-subunits, and these subunits can combine to form 24 distinct α/β heterodimers [83]. Each heterodimer exhibits unique but overlapping specificity for multiple ECM ligands.

Integrins serve as signaling hubs between the ECM and cells [84]. In diseased hearts, their expression and function are altered [85]. For example, myocardial stress-induced integrin signaling may lead to myofibroblast activation [37]. Integrin α5β1, a key fibronectin receptor for cell migration, induces conformational changes upon binding, triggering the FAK (Focal Adhesion Kinase)/Src (Src Proto-Oncogene Tyrosine Kinase) signaling pathway to drive fibroblast activation [86]. Binding of the laminin system to integrin α6β1 participates in transmitting myocardial contractile force to the extracellular matrix [87].

#### 6.2.2 Wnt

In myocardial fibrosis, Wnt ligands bind to cell membrane receptors and co-receptors LRP5/6 (Low-Density Lipoprotein Receptor-Related Protein 5/6), inhibiting the activity of the β-catenin degradation complex. This leads to stable accumulation of β-catenin in the cytoplasm and its translocation to the nucleus, which activates the expression of fibrosis-related genes and promotes the transformation of fibroblasts to myofibroblasts, and participates in the development of myocardial fibrosis. LRP6, as a co-receptor for Wnt ligands [88], promotes the upregulation of Wnt2 and Wnt4 to activate β-catenin/NF-κB signaling upon binding to Wnt [89], contributing to myocardial fibrosis. The stiff matrix after fibrosis upregulates Wnt5a through YAP/TAZ, activating the non-canonical Wnt signaling to promote fibroblast migration and collagen deposition [90]. Biglycan also affects Wnt-induced β-catenin-mediated transcriptional activity through interaction with LRP6 [91].

#### 6.2.3 TLR4

TLR has important functions in both innate and adaptive immunity, and also plays a role in the pathophysiology of various cardiovascular diseases. The knockout or loss of function of TLR4 can attenuate inflammatory injury, myocardial fibrosis, and apoptosis in various cardiovascular diseases [92, 93, 94, 95]. Upon ligand binding, TLR4 recruits MyD88, which thereafter triggers the activation of NF-κB and MAPK while facilitating the secretion of proinflammatory cytokines.

TLR4 can have numerous ECM proteins acting as its ligands. For example, TNC is an activator of TLR4 that regulates M1/M2 macrophage polarization through binding [72], upregulates IL-6 expression in human fibroblasts [96], and in damaged tissues, TLR4 signaling induces TNC expression [97], creating a vicious cycle. Intact FN participates in cell adhesion and repair, but its degradation fragments in response to inflammatory or oxidative stress (e.g., Fibronectin containing Extra Domain A) can act as damage-associated molecular patterns (DAMPs), activating the NF-κB signaling pathway via TLR4 to induce the release of inflammatory factors such as IL-1β and IL-6 [98, 99].

### 6.3 Activation of TGF-β1

TGF-β1 is a critical factor in myocardial fibrosis, and various components of the fibrogenic niche play roles in mediating its specific activation. For example, TGF-β binding proteins (LTBP) in the ECM form cross-links with fibrillins, which do not alter the interaction between latent TGF-β and integrin αVβ6 but enhance TGF-β activation [100]. Since LTBP is covalently tethered to the matrix through fibrillin, the matrix acts as a fixed “substrate” that spatially restricts the activation of downstream TGF-β pathways. Studies have shown that TSP-1 regulates TGF-β1 activation [101], and connective tissue growth factor (CTGF) also participates in TGF-β1 activation, inhibiting cardiac fibrosis by suppressing the TGF-β/Smad signaling pathway and modulating cardiac inflammation [102]. Changes in ECM stiffness can also activate the NF-κB pathway, enhancing IL-1β secretion, which further induces MMP-9 expression and TGF-β1 release. Previous studies have shown that the integrins αvβ6 and αvβ8 are crucial for the activation of TGF-β1 [103].

Thus, through binding to integrins, TLR4, and Wnt, as well as activation of TGF-β1, fibrogenic niche-resident ECM proteins eventually provoke the activation of NF-κB, β-catenin, and Smad3 to promote fibrosis. Furthermore, the TGF-β, YAP/TAZ, and Wnt/β-catenin pathways have been demonstrated to form a cross-regulatory network during the fibrotic process through molecular phosphorylation, ubiquitination, changes in subcellular localization, and nuclear transcription. This network participates in determining the fate of cells and in pathological processes [104].

### 6.4 Generation of Matricellular Factors

ECM components are constantly changing and remodeling during myocardial injury [105]. This remodeling may generate key factors that regulate cell activity. However, as data on the proteins produced in the fibrogenic niche are limited, further research is needed to investigate their potential roles in myocardial fibrosis. The in-depth exploration of the functional roles of stromal cytokines in the fibrogenic niche can serve as a novel research direction in the future.

## 7. Conclusions

The notion of the fibrogenic niche stands as quite distinct from current perspectives on the onset and progression of myocardial fibrosis. While the conventional view emphasized the significance of specific signaling pathways alongside cell types, the fibrogenic niche hypothesis centers on the establishment and progression of a pro-fibrotic microenvironment in the aftermath of tissue injury. A deeper comprehension of the fibrotic ecological niche may aid in the early diagnosis of the disease and support the advancement of individualized medicine.

ECM components and signaling pathway molecules may serve as non-invasive biomarkers for the early diagnosis of myocardial fibrosis. For example, LOX, which directly participates in ECM protein cross-linking, reflects structural and functional abnormalities of the ECM through changes in its activity, offering certain advantages for early diagnosis. Combining LOX with other cardiac-specific markers (e.g., troponin, NT-proBNP, etc.) can improve diagnostic specificity.

Based on this concept, we can develop new therapeutic approaches for treating myocardial fibrosis. For instance, we can implement a multi-targeted strategy to intervene in the progression of fibrosis by: (1) blocking mechanical transmission by weakening ECM fiber cross-linking and reducing ECM rigidity, and (2) inhibiting fibroblast activation by targeting known signaling pathways. Future challenges include addressing issues related to personalized treatment and long-term safety.
